# Neurological symptoms after COVID-19 vaccination: a report on the clinical presentation of the first 50 patients

**DOI:** 10.1007/s00415-023-11895-9

**Published:** 2023-07-29

**Authors:** Ameli Gerhard, Vanessa Raeder, Helena Franziska Pernice, Fabian Boesl, Maria Schroeder, Jonathan Richter, Matthias Endres, Harald Prüß, Katrin Hahn, Heinrich J. Audebert, Christiana Franke

**Affiliations:** 1 Department of Neurology and Experimental Neurology, Charité–Universitätsmedizin Berlin, Corporate Member of Freie Universität Berlin and Humboldt-Universität Zu Berlin, Berlin, Germany; 2 Center for Stroke Research Berlin, Berlin, Germany; 3 German Center for Neurodegenerative Diseases (DZNE), Berlin, Germany; 4 Excellence Cluster NeuroCure, Berlin, Germany; 5 German Center for Cardiovascular Research (DZHK), Berlin, Germany; 6 Berlin Institute of Health (BIH), Berlin, Germany

**Keywords:** Post-COVID-19 syndrome (PCS), SARS-CoV-2 vaccination, Post-COVID-19 vaccine syndrome (PVS), Paresthesia, Fatigue, Cognitive impairment

## Abstract

**Objectives:**

Neurological symptoms associated with Severe Acute Respiratory Syndrome Coronavirus-2 (SARS-CoV-2) vaccination were discovered in the context of billions of administered vaccine doses. The clinical manifestations often resemble post Coronavirus Disease 2019 (post-COVID-19) syndrome (PCS) features and may be considered as post-COVID-19 vaccine syndrome (PVS). Data regarding frequency, severity and pathophysiological mechanisms are scarce.

**Methods:**

We assessed routine clinical examinations in 50 patients reporting new-onset neurological symptoms after SARS-CoV-2 vaccination, including neurological examination, laboratory and electrophysiology tests, as well as self-report questionnaires measuring fatigue, depressive symptoms, anxiety, risk of somatic symptom disorder, and health-related quality of life. Patients were included when symptoms occurred after confirmed COVID-19 vaccination and without prior SARS-CoV-2 infection, and if no alternative diagnosis was found to explain the symptoms.

**Results:**

The most frequently reported symptoms were paraesthesia (56%), fatigue (46%) and cognitive impairment (36%). Neurological, routine laboratory, and electrophysiological examinations did not yield distinct pathological findings. Neuropsychological testing of a subgroup revealed deficits in attention, executive function and memory.

**Discussion:**

The spectrum of clinical manifestations post-vaccination poses a substantial overlap with PCS symptoms. As no pathological findings were obtained in routine diagnostics, uncertainty remains about the underlying pathophysiological mechanisms and requires further investigation beyond routine work-up.

## Introduction

Post-COVID-19 syndrome (PCS) is known for its wide range of neurological and psychiatric symptoms, including fatigue, cognitive impairment, headache, depression, and anxiety [3, 4, 19]. Vaccine development has been a major breakthrough not only to reduce the number of deaths and severe cases of Coronavirus Disease 2019 (COVID-19), but also to contain the spread of Severe Acute Respiratory Syndrome Coronavirus 2 (SARS-CoV-2) [6]. Additionally, there is emerging evidence that vaccination decreases the risk of developing PCS after breakthrough infection [1].

Initially, all approved vaccines were found to be safe, with generally mild and transient side effects such as pain on the injection site, fever, or headache [17]. A serious adverse event after vector vaccination is vaccine-induced immune thrombotic thrombocytopenia (VITT) [7], leading to predominant usage of mRNA-based vaccines in Germany. Other severe neurological adverse events following COVID-19 vaccination have been rarely described and involve e.g., Bell’s palsy, Guillain-Barré syndrome, acute disseminated encephalomyelitis, and cerebrovascular events [16]. Data on other symptoms in correlation with COVID-19 vaccination, commonly referred to as “post-COVID-19 vaccine syndrome” (PVS) [10], are scarce.

Here we report of 50 patients who presented to our neurological outpatient clinic due to neurological symptoms in temporal association to COVID-19 vaccination.

## Methods

Fifty patients with neurological symptoms following COVID-19 vaccination presented to our outpatient clinic between October 2021 and July 2022. They were interviewed and examined by a neurologist. All patients had received at least one vaccination against SARS-CoV-2 and did not report of a concomitant disease explaining the symptoms. Patients with a confirmed SARS-CoV-2 infection before symptom onset were excluded. Diagnostic evaluation including blood tests was performed according to the guidelines of the German Society of Neurology. Patients filled out self-report questionnaires regarding fatigue (Fatigue Severity Scale, FSS) [11], depression (Beck Depression Inventory Version I, BDI) [2], anxiety (Generalized Anxiety Disorder Scale-7, GAD-7) [18], and risk of somatic symptom disorder (Somatic Symptom Disorder – B Criteria Scale, SSD-12) [21]. Health-related quality of life was assessed using the Short-Form-36 Health Survey (SF-36) [23] and compared with a norm sample (n = 2471) [8]. To screen for cognitive deficits, the Montreal Cognitive Assessment Scale (MoCA) [15] was performed. Neuropsychological examination covered test items regarding learning and memory, complex attention, executive functions, language, and perceptual motor function.

Electrophysiological examination was conducted in patients with paraesthesia and neuropathic pain. Skin biopsy was performed to assess intraepidermal nerve fibre density or pathological amyloid deposits in those patients whose electrophysiological assessment did not show pathological findings.

## Results

The mean age of patients was 41 years (21–62 years) and the majority were female (60%). In 50% of patients, symptoms occurred after the first vaccination, in 26% after the second, and in 24% after the third (Table 1). Almost all patients (98%) developed symptoms after administration of an mRNA-based vaccine. Most patients (86%) received a homologous, mRNA-based vaccine regimen (82% BioNTech-Pfizer, 4% Moderna). Heterologous vaccine regimen were used in 14% of patients (10% mRNA-/mRNA-based, 4% vector-/mRNA-based). The median latency between receiving the vaccination and onset of symptoms was three days, ranging from one hour to 30 days. A SARS-CoV-2 infection occurred in 16 patients (32%) after their vaccination and onset of reported symptoms. All 16 had a mild course, but 10 (63%) reported an exacerbation of pre-existing symptoms.

**Table 1:** Patient characteristics and details of vaccine regimens

	Total	Female	Male
Number of patients	50	30 (60%)	20 (40%)
Mean age (years)	41.0	41.8	39.9
Range	21–62	26–62	21–60
Onset of symptoms after vaccine number			
1	25 (50%)	14 (47%)	11 (55%)
2	13 (26%)	8 (27%)	5 (25%)
3	12 (24%)	8 (27%)	4 (20%)
Onset of symptoms after vaccine type			
mRNA	49 (98%)	30 (100%)	19 (95%)
BioNTech-Pfizer	44 (88%)	27 (90%)	17 (85%)
Moderna	5 (10%)	3 (10%)	2 (11%)
Vector	1 (2%)	0 (0%)	1 (5%)
Johnson & Johnson	1 (2%)	0 (0%)	1 (5%)
AstraZeneca	0 (0%)	0 (0%)	0 (0%)
Vaccine regimen			
Homologous	43 (86%)	27 (90%)	16 (80%)
mRNA	43 (86%)	27 (90%)	16 (80%)
BioNTech-Pfizer	41 (82%)	26 (87%)	15 (75%)
Moderna	2 (4%)	1 (3%)	1 (5%)
Vector	0 (0%)	0 (0%)	0 (0%)
Heterologous	7 (14%)	3 (10%)	4 (20%)
mRNA/mRNA	5 (10%)	3 (10%)	2 (10%)
BioNTech-Pfizer/Moderna	5 (10%)	3 (10%)	2 (10%)
Vector/mRNA	2 (4%)	0 (0%)	2 (10%)
Johnson & Johnson/BioNTech-Pfizer	1 (2%)	0 (0%)	1 (5%)
AstraZeneca/BioNTech-Pfizer	1 (2%)	0 (0%)	1 (5%)
mRNA/vector	0 (0%)	0 (0%)	0 (0%)
Median latency until onset of symptoms (days)	3	2	4
Range	0–30	0–30	0–28
SARS-CoV-2 infection after vaccination	16 (32%)	10 (33%)	6 (30%)
Increase in the severity of symptoms due to infection	10 (63%)	7 (70%)	3 (50%)

The most frequent self-reported central nervous symptoms were fatigue (n = 23; 46%), cognitive impairment (n = 18; 36%), and headache (n = 15; 30%). Peripheral nervous symptoms included paraesthesia (n = 28; 56%), fasciculations (n = 11; 22%), myalgia (n = 11; 22%), and neuropathic pain (n = 11; 22%). Other symptoms included vertigo (n = 8; 16%) and tinnitus (n = 3; 6%). Overall, 32% presented with central symptoms, 40% with peripheral symptoms and 28% with both central and peripheral symptoms (Table 2). At the point of presentation in our outpatient clinic, none of the patients reported of ameliorated symptoms over time.

**Table 2:** Reported symptoms

	Total	Female	Male
Symptoms			
Fatigue	23 (46%)	13 (43%)	10 (50%)
Cognitive impairment	18 (36%)	11 (37%)	7 (35%)
Headache	15 (30%)	10 (33%)	5 (25%)
Paraesthesia	28 (56%)	17 (57%)	11 (55%)
Fasciculations	11 (22%)	6 (20%)	5 (25%)
Myalgia	11 (22%)	6 (20%)	5 (25%)
Neuropathic pain	11 (22%)	7 (23%)	4 (20%)
Vertigo	8 (16%)	7 (23%)	1 (5%)
Tinnitus	5 (10%)	2 (7%)	3 (15%)
Central	16 (32%)	10 (33%)	6 (30%)
Peripheral	20 (40%)	13 (43%)	7 (35%)
Both	14 (28%)	7 (23%)	7 (35%)

Of all 43 patients examined with MoCA, 40% showed pathologic scores (≤ 25/30 points). More comprehensive neuropsychological examination in 8 patients revealed deficits in attention (n = 6, 75%), executive function (n = 4, 50%) and memory (n = 3, 38%).

Brain MR imaging (n = 35) showed unremarkable findings except for unspecific gliosis (n = 3; 9%) and chronic mastoiditis (n = 1; 3%). Electroencephalography (n = 9) revealed no pathological findings. Electrophysiology (n = 27) detected single fasciculation potentials (n = 2; 7%) and bilateral carpal tunnel syndrome (n = 1; 4%). Skin biopsy was performed in four patients, which revealed normal intraepidermal nerve fibre density and no evidence of pathological amyloid deposits [12]. A routine serological examination including full blood count, liver enzymes, renal function, and electrolytes was carried out in all patients without detection of relevant pathological findings.

Substantial impairment due to fatigue quantified by FSS (≥ 4 points) was reported by 73% of patients (Table 3). BDI indicated moderate to severe depressive symptoms in 16% (≥ 20 points), and GAD-7 pointed to symptoms of moderate to severe anxiety in 25% (≥ 10 points) of patients. Evaluation by SSD-12 revealed an increased risk for somatic symptom disorder (≥ 23 points) in 75%. SF-36 scores were significantly worse (p < 0.05) in all domains in the post-vaccination group (physical functioning 59 vs 71%, physical pain 32 vs. 71%, role limitations due to physical health problems 14 vs. 53%, role limitations due to personal or emotional problems 52 vs. 66%, emotional well-being 54 vs. 70%, social functioning 38 vs. 79%, energy/fatigue 28 vs. 52%, general health perception 38 vs. 57%). No significant differences by gender were found in SF-36 results.

**Table 3:** Evaluation of self-questionnaires and results of Montreal cognitive assessment scale

		Total	Female	Male
*FSS*	Mean	5.1	5.3	4.9
(n = 45 (f n = 28, m n = 17), 1–7 points	No impairment due to fatigue (1–3 points)	12 (27%)	7 (25%)	5 (29%)
	Impairment due to fatigue (4–7 points)	33 (73%)	21 (75%)	12 (71%)
*BDI I*	Mean	13.2	14.3	11.4
n = 43 (f n = 27, m n = 16), 0–63 points	No depression (0–9 points)	15 (35%)	8 (30%)	7 (44%)
	Mild depression (10–19 points)	21 (49%)	13 (49%)	8 (50%)
	Moderate depression (20–29 points)	6 (14%)	5 (19%)	1 (6%)
	Severe depression (30–63 points)	1 (2%)	1 (4%)	0 (0%)
*GAD-7*	Mean	6.8	6.3	7.6
n = 44 (f n = 27, m n = 17), 0–21 points	Minimal anxiety (0–4 points)	17 (39%)	12 (44%)	5 (29%)
	Mild anxiety (5–9 points)	16 (36%)	8 (30%)	8 (47%)
	Moderate anxiety (10–14 points)	8 (18%)	6 (22%)	2 (12%)
	Severe anxiety (15–21 points)	3 (7%)	1 (4%)	2 (12%)
*SSD-12*	Mean	26.3	25.3	27.7
n = 44 (f n = 27, m n = 17), 0–48 points	No risk for somatic symptom disorder (0–22 points)	11 (25%)	8 (30%)	3 (18%)
	Risk for somatic symptom disorder (23–48 points)	33 (75%)	19 (70%)	14 (82%)
*MoCA*	Mean	25.7	25.7	25.8
n = 43 (f n = 26, m n = 17), 0–30 points	Normal (26–30 points)	26 (61%)	18 (69%)	8 (47%)
	Pathologic (≤ 25 points)	17 (40%)	8 (31%)	9 (53%)

*BDI* Beck’s depression inventory; *f* female, *FSS* fatigue severity scale; *GAD-7* generalized anxiety disorder 7; *m* male, *MoCA* Montreal cognitive assessment; *SSD-12* somatic symptom disorder—B criteria scale

## Discussion

We report of a comprehensive neurological assessment in 50 patients with persistent neurological symptoms in temporal relation to COVID-19 vaccination including clinical presentation, routine diagnostic, and self-report questionnaires. To date and in contrast to PCS, reliable data and a generally accepted definition of PVS are absent.

Our patients presented with central (cognitive impairment, fatigue, headache) and peripheral (paraesthesia, myalgia, fasciculations, neuropathic pain) neurological symptoms. Our data indicate that the spectrum of symptoms reported by PVS patients is similar to the spectrum we observe in PCS patients [3]. Notably, paraesthesia (56%) as the most common reported symptom in PVS was less frequently observed in PCS, while fatigue (46%) and cognitive impairment (36%) seem to occur more often in PCS [3]. As a limitation, our study did not include follow-up examinations. These are needed to assess the time course of symptom severity, modification and potential recovery of symptoms.

Self-report questionnaires showed an increased risk of somatic symptom disorder as assessed by SSD-12 in 75% of our 50 patients, moderate to severe anxiety measured by GAD-7 was present in 25%, and BDI indicated moderate to severe depressive symptoms in 16% of our patients. SF-36 showed low percentages in all scores, especially regarding physical health, underlining the severe impact on patient’s quality of live. Since standard diagnostic was widely normal, inclusion of psychiatric and psychosomatic assessment should be considered.

Our cohort mainly involved patients vaccinated with mRNA vaccines, which are predominantly administered in Germany. Available literature however accuses vector-based vaccines to be more frequently associated with (severe) neurological side effects [7, 20]. On the other hand, mRNA-COVID‐19 vaccine side‐effects have been attributed to a nocebo component in association with vaccine hesitancy [9].

Our data does not allow conclusions whether the symptoms occurred in temporal relationship to the COVID-19 vaccination, or whether the vaccine may be considered as a triggering factor or a cause of these symptoms. While there is limited data, several pathomechanisms have been suggested to explain the occurrence of diseases following SARS-CoV-2 vaccination, e.g., molecular mimicry, production of cross-reactive (anti-idiotype) autoantibodies, involvement of vaccine adjuvants, and persistence of spike protein [5, 14, 22].

Although neurological symptoms after COVID-19 vaccination have been reported, they appear to be rare considering the total number of vaccines administered. Current recommendations and guidelines favour COVID-19 vaccination as the risk of neurological complications during and after COVID-19 infection overweighs [13, 16]. Nevertheless, in-depth investigation of the individual patient, including diagnostic procedures beyond routine clinical care is needed, while research on pathophysiological mechanisms may provide further insights and might offer therapeutic options.

## Data Availability

The original contributions presented in the study are included in the article, further inquiries can be directed to the corresponding author.
